# Supplementary material for: A UMLS-based spell checker for natural language processing in vaccine safety
Source: BMC Med Inform Decis Mak. 2007 Feb 12;7:3. doi: 10.1186/1472-6947-7-3 (PMC1805499; doi:10.1186/1472-6947-7-3)
Supplement: Additional file 13 — RAP application directory. Sets up directory for RAP (RDF application for PHP) source code [file 1472-6947-7-3-S13.gz › rap/doc/phpdoc/classtrees_model.html]

xml version="1.0" encoding="iso-8859-1"?


## Root class DbStore

- Object **(Different package)**
  - DbStore

## Root class Model

- Object **(Different package)**
  - Model
    - DbModel
      - NamedGraphDb
  - MemModel
    - InfModel
      - InfModelB
      - InfModelF
    - NamedGraphMem

## Root class ModelFactory

- ModelFactory

## Root class Node

- Object **(Different package)**
  - Node
    - Literal
      - ResLiteral
  - Resource
    - BlankNode
    - ResResource
      - OntResource
        - Individual
        - OntClass
        - OntProperty
      - ResContainer
        - ResAlt
        - ResBag
        - ResSeq
      - ResList
      - ResProperty

## Root class Statement

- Object **(Different package)**
  - Statement
    - InfStatement

Documentation generated on Thu, 7 Jul 2005 13:41:07 +0200 by phpDocumentor 1.3.0RC3
